# pH-Responsive Cellulose/Silk/Fe_3_O_4_ Hydrogel Microbeads Designed for Biomedical Applications

**DOI:** 10.3390/gels10030200

**Published:** 2024-03-16

**Authors:** Seung Hyeon Weon, Yuhyeon Na, Jiwoo Han, Jeong Woo Lee, Hyung Joo Kim, Saerom Park, Sang Hyun Lee

**Affiliations:** Department of Biological Engineering, Konkuk University, Seoul 05029, Republic of Korea; shweon99@gmail.com (S.H.W.); nyh400@konkuk.ac.kr (Y.N.); jw991021@naver.com (J.H.); moon4737@naver.com (J.W.L.); hyungkim@konkuk.ac.kr (H.J.K.)

**Keywords:** cellulose, silk, microbead, pH responsive, protein support

## Abstract

In this study, cellulose/Fe_3_O_4_ hydrogel microbeads were prepared through the sol–gel transition of a solvent-in-oil emulsion using various cellulose-dissolving solvents and soybean oil without surfactants. Particularly, 40% tetrabutylammonium hydroxide (TBAH) and 40% tetrabutylphosphonium hydroxide (TBPH) dissolved cellulose at room temperature and effectively dispersed Fe_3_O_4_, forming cellulose/Fe_3_O_4_ microbeads with an average diameter of ~15 µm. Additionally, these solvents co-dissolved cellulose and silk, allowing for the manufacture of cellulose/silk/Fe_3_O_4_ hydrogel microbeads with altered surface characteristics. Owing to the negatively charged surface characteristics, the adsorption capacity of the cellulose/silk/Fe_3_O_4_ microbeads for the cationic dye crystal violet was >10 times higher than that of the cellulose/Fe_3_O_4_ microbeads. When prepared with TBAH, the initial adsorption rate of bovine serum albumin (BSA) on the cellulose/silk/Fe_3_O_4_ microbeads was 18.1 times higher than that on the cellulose/Fe_3_O_4_ microbeads. When preparing TBPH, the equilibrium adsorption capacity of the cellulose/silk/Fe_3_O_4_ microbeads for BSA (1.6 g/g) was 8.5 times higher than that of the cellulose/Fe_3_O_4_ microbeads. The pH-dependent BSA release from the cellulose/silk/Fe_3_O_4_ microbeads prepared with TBPH revealed 6.1-fold slower initial desorption rates and 5.2-fold lower desorption amounts at pH 2.2 than those at pH 7.4. Cytotoxicity tests on the cellulose and cellulose/silk composites regenerated with TBAH and TBPH yielded nontoxic results. Therefore, cellulose/silk/Fe_3_O_4_ microbeads are considered suitable pH-responsive supports for orally administered protein pharmaceuticals.

## 1. Introduction

Hydrogels are composed of interlinked hydrophilic polymers that form three-dimensional networks. Chemical or physical crosslinking or polymerization assists in making the structure insoluble in water while retaining a significant amount of water when it is swollen [1,2]. Biopolymer-based hydrogels derived from natural resources not only possess high water retention capacities but are also biocompatible, biodegradable, nontoxic, and affordable [3]. The biopolymers mainly used to create hydrogels include cellulose, alginate, agarose, chitosan, and carrageenan [4].

Cellulose is the most abundant biopolymer in nature and the most prevalent and investigated renewable material [5]. The three free hydroxyl groups of cellulose form hydrogen bonds, resulting in a highly crystalline structure that renders it insoluble in water. This stable crystalline structure contributes to its excellent mechanical strength and thermal resistance [6,7]. The structural functionality and eco-friendly properties of cellulose hydrogels demonstrate their excellent potential in the field of biotechnology, including in drug delivery systems, tissue-engineering scaffolds, cell cultures, enzyme immobilization, and water purification [8,9,10]. To create a hydrogel using unmodified cellulose, the selection of a solvent that can effectively dissolve and regenerate cellulose is crucial. With the recent development of various solvents capable of dissolving cellulose, there is growing interest in cellulose hydrogels. To overcome the high cost and toxicity issues associated with traditionally used solvents like *N*-methylmorpholine-*N*-oxide or LiCl/dimethylacetamide [11,12], non-volatile ionic liquids (ILs) such as 1-ethyl-3-methylimidazolium acetate ([Emim][Ac]) or 1-butyl-3-methylimidazolium chloride ([Bmim][Cl]) have been developed [13]. More recently, affordable solvents such as alkylammonium hydroxide and alkylphosphonium hydroxide have been introduced [11].

Micrometer-sized cellulose hydrogel beads have potential applications in various fields, such as drug delivery, chromatography, and dye or metal ion adsorption, and as enzyme supports [7,14]. Cellulose hydrogel microbeads are created through a sol–gel transition process involving the physical dissolution of cellulose in a solvent, the formation of a cellulose solution-in-oil emulsion, and subsequent regeneration using antisolvents [11]. The regularity and diameter of the cellulose hydrogel microbeads can be determined by several factors, including the concentration and viscosity of the cellulose solution, ratio of oil to cellulose solution, type and amount of surfactant used, and stirring speed and temperature [15,16].

Blending biopolymers with cellulose can conveniently control the physical and chemical properties of cellulose hydrogels. The interpenetrating polymer networks formed through this method exhibit enhanced mechanical performances and unique properties, such as high strength and toughness, self-healing, and antimicrobial capabilities [17]. The development of solvents capable of simultaneously dissolving two or more biopolymers is crucial for creating biopolymer blends. Owing to the strong biopolymer-dissolving capabilities of ILs, diverse materials based on blended biopolymers can be produced. Liu et al. and Peng et al. prepared cellulose/chitosan/Fe_3_O_4_ hydrogel microbeads as enzyme-immobilizing supports and Cu^2+^ adsorbents via sol–gel transition using [Bmim][Cl] [18,19]. Our group previously prepared various blended cellulose/biopolymer hydrogel microbeads, including cellulose/lignin/Fe_3_O_4_, cellulose/chitosan/Fe_3_O_4_, cellulose/starch/Fe_3_O_4_, and cellulose/carrageenan/Fe_3_O_4_, for dye and protein adsorption [14]. We also prepared photocatalytic hydrogel microbeads, such as cellulose/carrageenan/TiO_2_ and cellulose/chitosan/TiO_2_, through a sol–gel transition using [Emim][Ac] [20].

Stimulus-sensitive hydrogels offer many possibilities in biotechnology because they exhibit “intelligent” behavior primarily through noncovalent dynamic bonding, such as hydrogen bonding, hydrophobic interactions, π-π stacking, and electrostatic interactions [21]. The pH of the surrounding environment is crucial from a biomedical perspective because the human body experiences pH changes in the gastrointestinal tract, certain tissues, tumor sites, and subcellular compartments. pH-sensitive hydrogels are typically produced by incorporating acidic or basic functional groups into a polymer backbone [22]. These groups either accept or release protons, leading to significant volume changes in response to the pH and ionic strength in aqueous environments [23]. Silk has a variety of functional groups in its structure, such as amine and carboxyl groups, that enable its interactions with drugs or proteins. The carboxyl groups in silk tend to contract at acidic pH, making them suitable for pH-sensitive hydrogels. These silk-based, pH-sensitive hydrogels can deliver drugs through the gastrointestinal tract to the intestines or release drugs into the alkaline environment of an infected burn wound [24,25,26].

However, in order to develop cellulose/silk hydrogel microbeads, selecting a solvent that can simultaneously dissolve cellulose and silk is a challenging issue. Therefore, we aimed to propose a new method for manufacturing cellulose-based hydrogel microbeads by selecting the most suitable solvent for the fabrication of cellulose/silk hydrogel microbeads. Additionally, we attempted a method of manufacturing microbeads without the use of surfactants to form emulsions, which has the advantage of simplifying the purification process.

In this study, cellulose hydrogel microbeads were prepared through the sol–gel transition of a solvent-in-oil emulsion by employing various cellulose-dissolving solvents without the use of surfactants. Additionally, pH-responsive cellulose/silk/Fe_3_O_4_ hydrogel microbeads were produced by blending silk with cellulose. To understand the characteristics of the cellulose/silk/Fe_3_O_4_ hydrogel microbeads, their average diameter, surface morphology, swelling ratio, and dye adsorption properties were investigated. The potential application of these cellulose/silk/Fe_3_O_4_ hydrogel microbeads as pH-responsive supports for protein drug delivery was explored by studying the adsorption and release properties of a model protein, bovine serum albumin (BSA). Finally, the biocompatibility of the cellulose/silk composites was confirmed.

## 2. Results and Discussion

### 2.1. Preparation of Cellulose/Fe_3_O_4_ Hydrogel Microbeads with Various Cellulose Solvents

Cellulose (MCC, 4% *w*/*v*) could be dissolved in six different solvents: [Emim][Ac] and aqueous solutions of LiBr, ZnCl_2_, tetrabutylammonium hydroxide (TBAH), tetrabutylphosphonium hydroxide (TBPH), and NaOH/thiourea (TU). Magnetic particles (Fe_3_O_4_) were also well dispersed in these cellulose solutions. The cellulose/Fe_3_O_4_ hydrogel microbeads were successfully obtained through the ethanol-mediated sol–gel transition of a solvent-in-oil emulsion prepared without using a surfactant. The prepared hydrogel microbeads were easily recovered within 10 min using a neodymium magnet. The dissolution conditions for the cellulose and the average diameters of the prepared cellulose/Fe_3_O_4_ hydrogel microbeads are listed in Table 1. TBAH and TBPH rapidly dissolved cellulose at room temperature, whereas [Emim][Ac], LiBr, and ZnCl_2_ required high-temperature conditions and NaOH/TU required low-temperature conditions for cellulose dissolution. Additionally, the average diameters of the cellulose/Fe_3_O_4_ hydrogel microbeads produced using TBAH and TBPH as solvents were significantly smaller, measuring 15 and 18 μm, respectively, compared to the hydrogel microbeads produced with the other solvents. Cellulose microbeads, used as a drug delivery system, have an advantage when a particle is as small as 10 µm. During oral administration, these microbeads penetrate the intestinal mucosa, potentially extending their residence time in the intestine [27]. A small particle size is essential for pulmonary delivery [28]. In this study, TBAH and TBPH were chosen as the solvents to create cellulose-based hydrogel microbeads. TBAH and TBPH effectively dissolved cellulose under mild conditions and exhibited the advantage of producing microbeads of approximately 10 μm, with good distribution and without the need for surfactants.

### 2.2. Preparation of Cellulose/Silk/Fe_3_O_4_ Hydrogel Microbeads

The physical and chemical properties of cellulose hydrogel microbeads can be conveniently modified by blending them with additional biopolymers. Silk is biocompatible with proteins and possesses biodegradable features, making it a commonly used medical material. When silk is combined with other hydrogel materials to form a composite, it alters the surface charge of the hydrogel to negative [29,30,31]. Hydrogel microbeads were manufactured by blending cellulose with silk. The silk, which was degummed by removing sericin, which can negatively affect the adsorption properties, co-dissolved well with cellulose in TBAH and TBPH. Subsequently, hydrogel microbeads composed of cellulose/silk/Fe_3_O_4_ were successfully manufactured using the same sol–gel transition method employed to produce the cellulose/Fe_3_O_4_ hydrogel microbeads.

**Figure 1:**
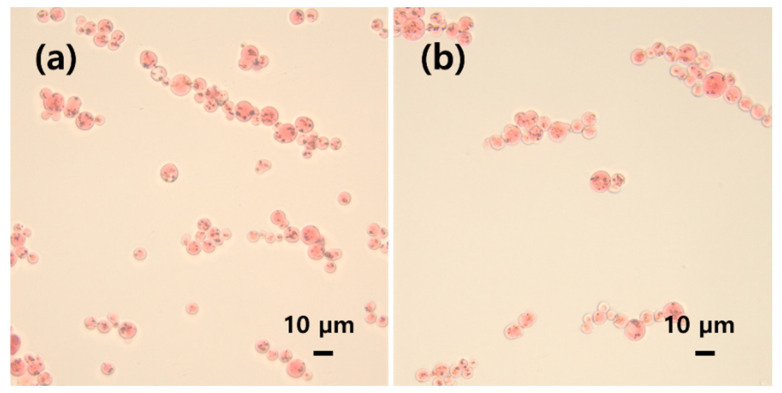
Optical microscopic images of cellulose/silk/Fe_3_O_4_ hydrogel microbeads prepared using TBAH (**a**) and TBPH (**b**). The contents of cellulose, silk, and Fe_3_O_4_ in the microbead-preparing solution were 4, 1, and 0.5%, respectively. Hydrogel microbeads were stained with Congo red.

**Figure 2:**
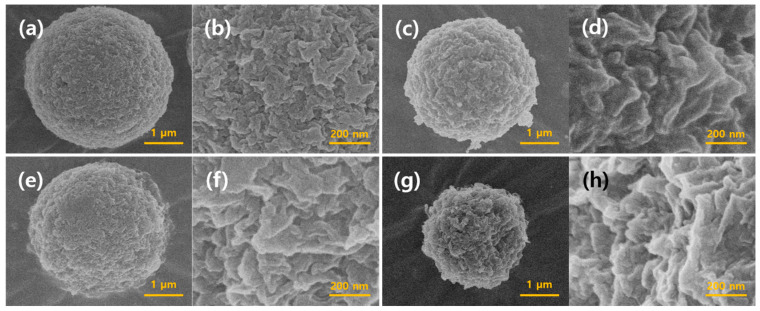
FE-SEM images of cellulose-based microbeads: (**a**,**b**) cellulose/Fe_3_O_4_ microbeads prepared using TBAH; (**c**,**d**) cellulose/silk/Fe_3_O_4_ microbeads prepared using TBAH; (**e**,**f**) cellulose/Fe_3_O_4_ microbeads prepared using TBPH; (**g**,**h**) cellulose/silk/Fe_3_O_4_ microbeads prepared using TBPH. The contents of cellulose, silk, and Fe_3_O_4_ in the microbead-preparing solution were 4, 1, and 0.5%, respectively.

Figure 1 shows the optical microscopy images of the cellulose/silk/Fe_3_O_4_ hydrogel microbeads prepared using TBAH and TBPH as solvents. The observed hydrogel microbeads were consistently spherical in shape, with Fe_3_O_4_ nanoparticles entrapped within the microbeads or exposed on their surfaces. Figure 2 shows FE-SEM images of the cellulose/Fe_3_O_4_ and cellulose/silk/Fe_3_O_4_ microbeads at the same magnification. All microbeads maintained their spherical shape, even after freeze drying. The surface roughness varied slightly depending on the solvent used, with the hydrogel microbeads prepared with TBPH exhibiting rougher surfaces than those prepared with TBAH. The cellulose/silk/Fe_3_O_4_ hydrogel microbeads appeared to shrink more after freeze drying than the cellulose/Fe_3_O_4_ hydrogel microbeads. In addition, the surfaces of the cellulose/silk/Fe_3_O_4_ microbeads were rougher than those of the cellulose/Fe_3_O_4_ microbeads.

During the blending of protein and polysaccharide, primary and secondary forces play a significant role in the formation and stability of protein–polysaccharide crystallites such as β-sheets and carbohydrate crystalline structures. The disordered β-turns of silk can interact with cellulose to form crystalline β-sheets, enhancing the mechanical strength of the hydrogel [32]. This can be seen in the β-sheet adsorption bands of 1618 cm^−1^ (amide I) and 1514 cm^−1^ (amide II) in the FT-IR spectra of the cellulose/silk composites (Figure S1). However, the amide peaks were weak in the cellulose/silk composites, and Liu et al. [33] and Park et al. [14] reported that distinguishing the peaks of biopolymers added to cellulose-based materials was challenging because of their weakness or overlap.

**Figure 3:**
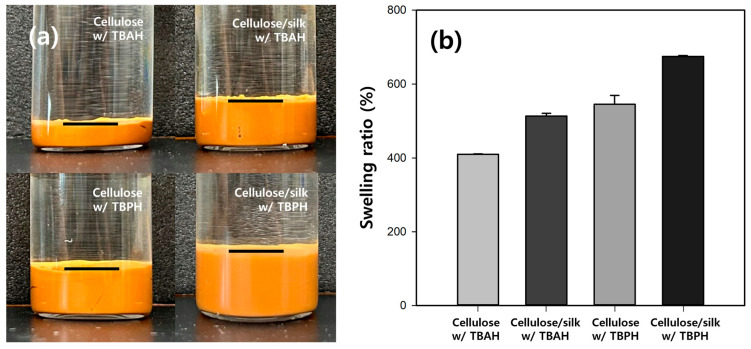
Swelling volumes (**a**) and swelling ratios (**b**) of cellulose-based hydrogel microbeads with same dry weight. The contents of cellulose, silk, and Fe_3_O_4_ in the microbead-preparing solution were 4, 1, and 0.5%, respectively.

To investigate the swelling properties of the cellulose/silk/Fe_3_O_4_ microbeads, we compared the volumes after adequately swelling the microbeads with the same dry weight (Figure 3a). The results showed that the volume of the swollen microbeads varied significantly depending on the dissolution solvent and the presence of silk. The volume of the cellulose/Fe_3_O_4_ hydrogel microbeads prepared with TBAH was the smallest, approximately half that of those produced with TBPH. The addition of silk notably increased the volume of the hydrogel microbeads, resulting in approximately 2- and 1.5-fold increases in the cellulose/silk/Fe_3_O_4_ hydrogel microbeads prepared with TBAH and TBPH, respectively. The swelling ratios of the microbeads were also measured after soaking them in distilled water for 48 h (Figure 3b). The swelling ratio of the cellulose/Fe_3_O_4_ microbeads prepared with TBPH was 1.3-fold higher than that of those prepared with TBAH. Silk significantly enhanced the swelling ratio, resulting in approximately 1.3 times higher swelling for both cellulose/silk/Fe_3_O_4_ microbeads prepared with TBAH and those prepared with TBPH. The swelling ratio of hydrogel microbeads is primarily determined by the functional groups on their surfaces [34]. A more negatively charged surface can enhance the electrostatic repulsion between the polymer chains or particles, preventing them from coming too close and allowing for greater water absorption. The presence of silk on the surface further negatively charged the hydrogel microbeads, and this increased surface negative charge can be considered responsible for the significantly higher swelling ratio of the cellulose/silk/Fe_3_O_4_ hydrogel microbeads compared with that of the cellulose/Fe_3_O_4_ hydrogel microbeads. Additionally, as shown in Figure 2, the surfaces of the cellulose/silk/Fe_3_O_4_ hydrogel microbeads were rougher than those of the cellulose/Fe_3_O_4_ hydrogel microbeads, indicating a larger surface area. This increased surface area can also be considered a contributing factor to the enhanced swelling ratio. The findings of this study indicate that cellulose/silk composites have a higher swelling ratio than cellulose, which is consistent with the results reported in previous studies [35,36,37].

To assess the storage stability of the cellulose/silk/Fe_3_O_4_ hydrogel microbeads, the amount of protein released after prolonged storage was measured. Initially, the quantity of silk present in the hydrogel microbeads was measured as the protein amount using a Micro BCA assay and set at 100%. After 20 days of storage at 25 °C, the protein released was less than 3% (Figure S2). The thermal stability of the cellulose/silk/Fe_3_O_4_ hydrogel microbeads was also assessed at various temperatures. The amount of leaked protein remained below 2% after 24 h of incubation at 45 °C. Even at 55 °C, the amount of protein released was very small, constituting less than 5% of the total protein (Figure S3). In addition, no protein digested by pepsin was detected at pH 2.2, indicating the high chemical stability of cellulose/silk/Fe_3_O_4_ hydrogel microbeads in simulated gastric fluid. The hydrogel microbeads prepared with TBAH were slightly more stable than those prepared with TBPH, although this difference was not statistically significant. These results indicate that the silk in the cellulose/silk/Fe_3_O_4_ hydrogel microbeads was tightly bound to the cellulose, preventing easy leakage, even under high temperatures, low pH, the presence of pepsin, and during extended storage periods.

### 2.3. Characteristics of Cellulose/Silk/Fe_3_O_4_ Hydrogel Microbeads with Various Silk Contents

**Figure 4:**
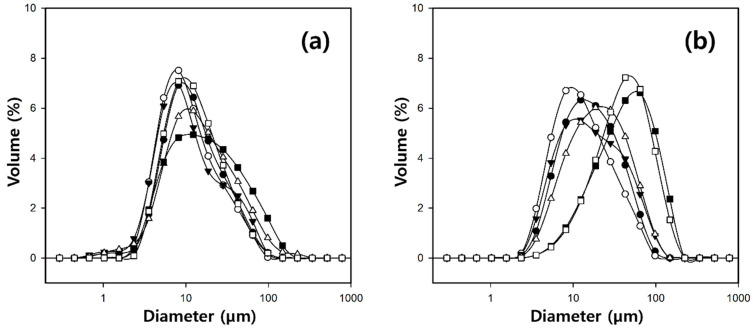
Size distribution of cellulose/silk/Fe_3_O_4_ hydrogel microbeads with various silk contents (●: 0%; ○: 0.25%; ▼: 0.5%; ∆: 1%; ■: 1.5%; and □: 2%) prepared using TBAH (**a**) and TBPH (**b**).

To investigate the characteristics of the microbeads produced by varying the silk concentrations, the cellulose/silk/Fe_3_O_4_ hydrogel microbeads were manufactured with a fixed cellulose concentration of 4% in the solvent, and the silk concentration was varied from 0 to 2%. The particle size distributions of the cellulose/silk/Fe_3_O_4_ hydrogel microbeads produced with various silk concentrations are shown in Figure 4. When TBAH was used as the solvent, the microbeads exhibited a wider size distribution as the silk concentration increased from 0 to 2%. However, the average particle size remained around 15 µm with no significant difference. Meanwhile, the microbeads produced with TBPH as the solvent also exhibited a constant average particle size of about 18 µm as the silk concentration increased from 0 to 1%. However, beyond a 1.5% silk concentration, there was a rapid increase in the particle size.

**Figure 5:**
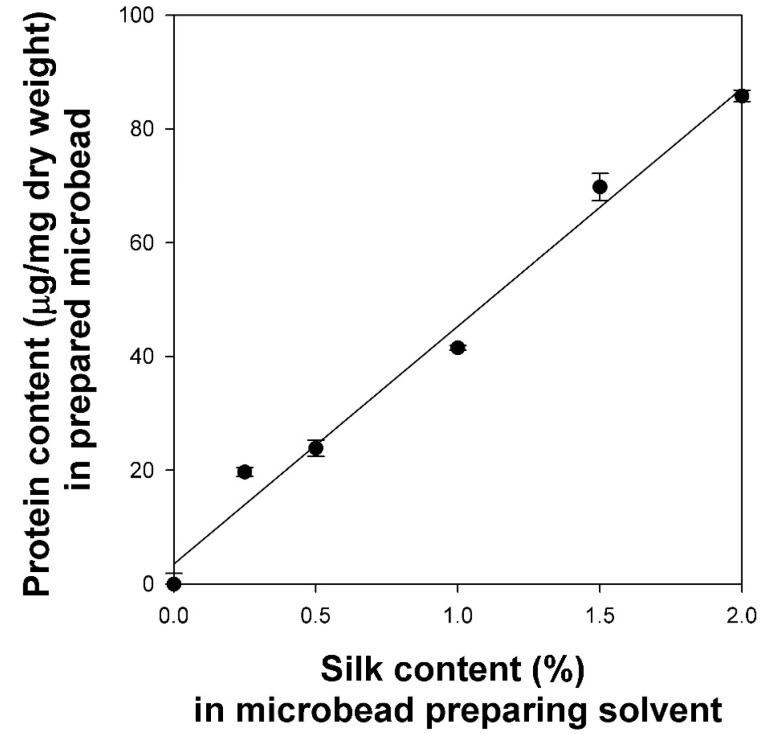
Protein contents in cellulose/silk/Fe_3_O_4_ hydrogel microbeads prepared with various silk contents. TBPH was used as the cellulose-dissolving solvent.

To determine the degree of surface modification of the cellulose/silk/Fe_3_O_4_ hydrogel microbeads based on the dissolved silk content in the solvent, the protein contents of the microbeads were measured using a Micro BCA assay (Figure 5). The cellulose/Fe_3_O_4_ microbeads were used as controls because the reducing ends of cellulose can also reduce the Cu^2+^ of bicinchoninic acid to Cu^+^, resulting in a violet color [38]. While this method has limitations in accurately quantifying the silk content within the microbead, it allows for the observation of changes in the silk concentration on the microbead surface. When the silk concentration in the solvent increased from 0 to 2%, the protein contents of the cellulose/silk/Fe_3_O_4_ hydrogel microbeads exhibited a linear increase with a high correlation coefficient (R^2^ = 0.986). These results demonstrate that even under the condition in which the silk concentration reaches 50% of the cellulose concentration, cellulose and silk can still strongly bind to each other, forming a three-dimensional network to compose the microbead.

**Figure 6:**
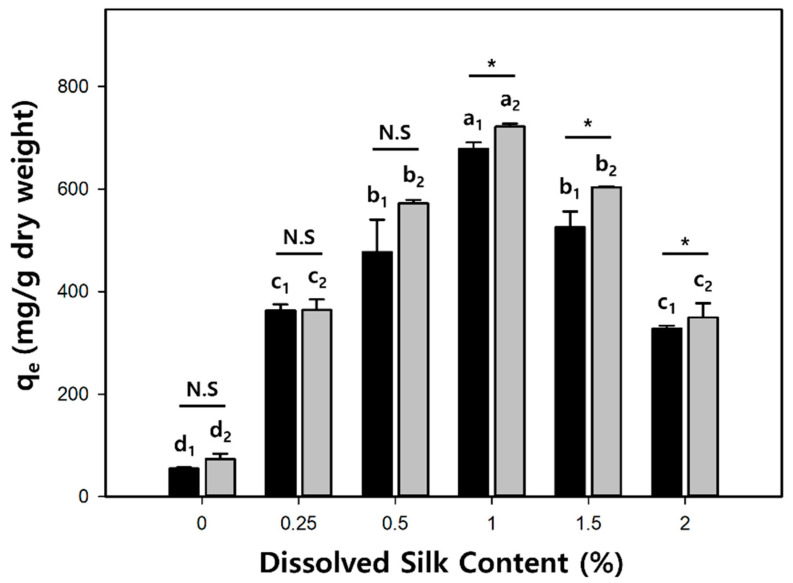
The effect of silk content in cellulose/silk/Fe_3_O_4_ hydrogel microbeads on the adsorption capacity for CV at pH 7. Black bars (TBAH) and gray bars (TBPH) represent the solvent used to prepare the microbeads. The initial concentration of CV for adsorption was 700 µg/mL. One-way analysis of variance (ANOVA) for silk content using Tukey’s test (*p* < 0.05); inset letters suggest the group classified from Tukey’s test. The subscripts (1, 2) are in the same group for the ANOVA test. The same letter indicates that there is no significant difference between the data. Asterisks (*) indicate statistical significance by *t*-test analysis of variance for the solvent (*p* < 0.05). N.S: not significant.

To investigate whether silk successfully altered the surface characteristics of the cellulose/Fe_3_O_4_ hydrogel microbeads, the adsorption properties of the cationic dye crystal violet (CV) on the cellulose/silk/Fe_3_O_4_ hydrogel microbeads with various silk contents were studied at pH 7 (Figure 6). Compared to cellulose/Fe_3_O_4_ hydrogel microbeads, the addition of just a small amount of silk (0.25%) resulted in approximately 6.6- and 5.0-fold higher adsorption capacities at equilibrium (q_e_) for the microbeads prepared with TBAH and TBPH, respectively. The increased adsorption capacity can be attributed to the enhancement of the electrostatic attraction between the positive charge of the CV and the negative charge of the cellulose/silk/Fe_3_O_4_ hydrogel microbeads. This result suggests that the surfaces of the cellulose/silk/Fe_3_O_4_ hydrogel microbeads underwent an overall change to a negative state when cellulose and silk were co-dissolved in the same solvent and subsequently regenerated as an interpenetrating polymer network to form microbeads [17,39]. Our group previously reported that cellulose/biopolymer films with lower zeta potentials exhibit higher CV adsorption abilities. Among these, silk, which has an isoelectric point of 4.1, has the lowest zeta potential. In addition, the cellulose/silk film exhibited a higher water contact angle than cellulose, indicating that its surface is more hydrophobic [36]. Therefore, electrostatic attraction and the hydrophobic effect could be the major adsorption mechanisms of CV on cellulose/silk/Fe_3_O_4_ hydrogel microbeads. As the dissolved silk content in the solvent increased, the q_e_ value of the cellulose/silk/Fe_3_O_4_ hydrogel microbeads exhibited an increasing trend, but after peaking at 1%, it decreased. In the case of the cellulose/silk/Fe_3_O_4_ hydrogel microbeads prepared with TBPH, there was a phenomenon where the average diameter sharply increased when the content of silk in the solvent was higher than 1.5% (Figure 4). As the particle size increased, the surface area per unit volume decreased, potentially leading to a reduction in the CV adsorption capacity. In the case of the cellulose/silk/Fe_3_O_4_ hydrogel microbeads prepared with TBAH, the particle size distribution tended to broaden when the solvent contained more than 1.5% silk. This suggests that the presence of larger particles may have reduced the CV adsorption capacity. The q_e_ values for the cellulose/silk/Fe_3_O_4_ hydrogel microbeads containing 1% silk in the solvent were 678 and 721 mg/g dry weight when prepared with TBAH and TBPH as the solvents, respectively, and these values were 12.4 and 9.8 times higher compared to those of the cellulose/Fe_3_O_4_ hydrogel microbeads, respectively. When the silk concentration in the solvent was 0.5% or lower, the CV adsorption capacity of the cellulose/silk/Fe_3_O_4_ hydrogel microbeads did not exhibit statistically significant differences based on the type of solvent used. However, when the silk concentration in the solvent was 1% or higher, the cellulose/silk/Fe_3_O_4_ hydrogel microbeads prepared with TBPH exhibited a significantly higher adsorption capacity compared to those prepared with TBAH (*p* < 0.05). This phenomenon is likely attributable to the rougher surfaces (Figure 2) and higher swelling ratios (Figure 3) of the cellulose/silk/Fe_3_O_4_ hydrogel microbeads prepared with TBPH than those prepared with TBAH [40]. These results demonstrate that the incorporation of silk into cellulose/Fe_3_O_4_ hydrogel microbeads markedly enhances the adsorption properties for CV by inducing a more negative surface. The optimal silk proportion for mixing with 4% cellulose was 1%, which yielded the highest q_e_ value. Therefore, subsequent experiments were performed using the corresponding ratios of the cellulose/silk/Fe_3_O_4_ hydrogel microbeads.

### 2.4. Kinetic Study of BSA Adsorption on Cellulose/Silk/Fe_3_O_4_ Hydrogel Microbeads

**Figure 7:**
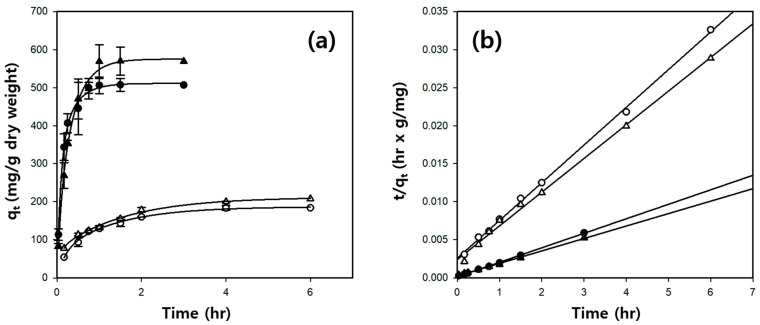
Effect of contact time (**a**) and pseudo-second-order model fitting (**b**) for BSA adsorption on cellulose/silk/Fe_3_O_4_ hydrogel microbeads. Filled and blanked symbols represent the cellulose/Fe_3_O_4_ microbeads prepared with and without silk, respectively. Circle symbols (TBAH) and triangle symbols (TBPH) represent the solvent used to prepare the microbeads. The initial concentrations of BSA were 100 and 500 µg/mL for microbeads without and with silk, respectively.

Figure 7a shows the amount of BSA, a model protein, adsorbed onto the hydrogel microbeads over time. The cellulose/Fe_3_O_4_ and cellulose/silk/Fe_3_O_4_ hydrogel microbeads reached equilibrium after 4 and 1 h, respectively. A pseudo-second-order kinetic model was used to investigate the BSA adsorption kinetics, which can be expressed by the following equation [41]:

$$\frac{t}{q_{t}}=\frac{1}{k_{2}q_{e}^{2}}+\frac{t}{q_{e}},$$

where k_2_ (g/mg·h) is the rate constant of the pseudo-second-order kinetic model, q_t_ (mg/g) is the amount of BSA adsorbed at time (t) (h), and q_e_ (mg/g) is the theoretical adsorption capacity, which can be calculated from the plot of t/q_t_ versus t in Figure 7b.

As shown in Table 2 and Figure 7b, both the cellulose/Fe_3_O_4_ and cellulose/silk/Fe_3_O_4_ hydrogel microbeads exhibit straight lines with high correlation coefficient values when represented by plots of t/q_t_ against t. In addition, the experimental q_e_ values are very similar to the theoretically calculated q_e_ value. Consequently, it was inferred that the BSA adsorption on the cellulose/Fe_3_O_4_ and cellulose/silk/Fe_3_O_4_ hydrogel microbeads was suitable for the pseudo-second-order kinetic model rather than the pseudo-first-order kinetic model (Table S1). Comparing the initial adsorption rates calculated as $k_{2}q_{e}^{2}$, the initial adsorption rate of the cellulose/silk/Fe_3_O_4_ hydrogel microbeads was much higher than that of the cellulose/Fe_3_O_4_ hydrogel microbeads. Specifically, when manufactured with TBAH, it was 18.1 times faster, and when manufactured with TBPH, it was 8.9 times faster. These results demonstrate that silk significantly enhances both the initial adsorption rate and the adsorption capacity of BSA. The initial adsorption rate onto and capacity of BSA in the cellulose/silk/Fe_3_O_4_ hydrogel microbeads were significantly influenced by the type of solvent used to prepare the microbeads. The initial adsorption rate of the cellulose/silk/Fe_3_O_4_ hydrogel microbeads for BSA was approximately 1.9 times higher when manufactured with TBAH than when manufactured with TBPH. However, when the adsorption equilibrium was reached, the q_e_ value of the cellulose/silk/Fe_3_O_4_ hydrogel microbeads for BSA was 1.2 times higher when they were manufactured with TBPH than that of those manufactured with TBAH.

### 2.5. Isotherm Study of BSA Adsorption on Cellulose/Silk/Fe_3_O_4_ Hydrogel Microbeads

**Figure 8:**
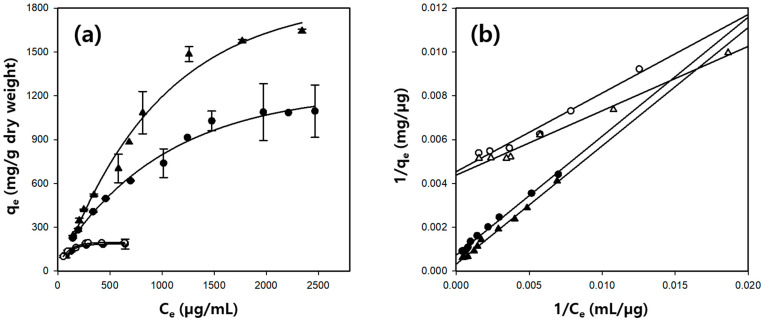
Effect of BSA concentration (**a**) and Langmuir model fitting as the best-fitted isotherm model (**b**) for BSA adsorption on cellulose/silk/Fe_3_O_4_ hydrogel microbeads. Filled and blanked symbols represent cellulose/Fe_3_O_4_ microbeads prepared with and without silk, respectively. Circle symbols (TBAH) and triangle symbols (TBPH) represent the solvents used to prepare the microbeads.

The adsorption isotherm study describes the equilibrium behavior of the adsorbent at a constant temperature. This requires sufficient contact between the adsorbate and adsorbent to reach a dynamic equilibrium with various concentrations of the adsorbate at a fixed adsorbent content. It is essential to obtain information about the maximum adsorption capacity of the adsorbent and the characteristics of the adsorption process [42]. Figure 8a shows the effect of the BSA concentration on the adsorption capacity of the cellulose-based hydrogel microbeads for BSA. For the cellulose/Fe_3_O_4_ hydrogel microbeads, adsorption was performed in BSA solutions with concentrations ranging from 50 to 650 µg/mL. The adsorption capacity (q_e_) increased with the increasing BSA concentration, reaching equilibrium at concentrations above 270 µg/mL. In the case of the cellulose/silk/Fe_3_O_4_ hydrogel microbeads, a wider range of BSA solutions with concentrations from 100 to 2500 µg/mL was prepared for adsorption. The adsorption capacity of the cellulose/silk/Fe_3_O_4_ hydrogel microbeads prepared with TBAH increased with the increasing BSA concentration and reached equilibrium at approximately 1500 µg/mL. In contrast, the cellulose/silk/Fe_3_O_4_ hydrogel microbeads prepared with TBPH reached equilibrium at a higher concentration of 2500 µg/mL. In the case of the cellulose/Fe_3_O_4_ hydrogel microbeads, there was no significant difference in the adsorption capacity for BSA depending on the type of solvent used for manufacturing. However, for the cellulose/silk/Fe_3_O_4_ hydrogel microbeads, the adsorption capacity for BSA varied significantly depending on the type of solvent used in the manufacturing process. When TBAH was used as the solvent for microbead production, the experimentally measured maximum q_e_ value for the cellulose/silk/Fe_3_O_4_ hydrogel microbeads was 1095 mg/g, which was 5.9 times higher than that for the cellulose/Fe_3_O_4_ hydrogel microbeads. Meanwhile, when TBPH was used as the solvent for microbead production, the experimentally measured maximum q_e_ value for the cellulose/silk/Fe_3_O_4_ hydrogel microbeads was 1643 mg/g, which was 8.5 times higher than that of the cellulose/Fe_3_O_4_ hydrogel microbeads. This is significantly higher compared to previously reported cellulose-based microbeads (Table S2) [43,44,45].

The Langmuir isotherm model provided the best fit for the investigated BSA adsorption isotherms compared with other models (Table S3). The Stum and Morgan equation is as follows [46]:

$$\frac{1}{q_{e}}=\frac{1}{q_{m}b}\frac{1}{C_{e}}+\frac{1}{q_{m}}‚$$

where C_e_ (µg/mL) is the BSA concentration in the solution at equilibrium; b (mL/µg) is the Langmuir constant; q_e_ (mg/g) is the amount of BSA adsorbed at equilibrium; and q_m_ (mg/g) is the theoretical maximum adsorption capacity. The Langmuir parameters for BSA adsorption are presented in Table 3. The correlation coefficients for the cellulose/silk/Fe_3_O_4_ hydrogel microbeads were high, exceeding 0.99. Hence, the cellulose/silk/Fe_3_O_4_ hydrogel microbeads adsorbed BSA onto their homogeneous surfaces via monolayer adsorption. The experimentally measured maximum q_e_ value of the cellulose/silk/Fe_3_O_4_ hydrogel microbeads prepared with TBAH was 1095 mg/g, and the q_m_ value calculated using the Langmuir model was 1378 mg/g, which was not significantly different. However, for the cellulose/silk/Fe_3_O_4_ hydrogel microbeads prepared with TBPH, the q_m_ value (3142 mg/g) was significantly higher than the experimentally measured maximum q_e_ value (1643 mg/g). This discrepancy is attributable to experimental errors caused by the excessively high adsorption capacity of the cellulose/silk/Fe_3_O_4_ hydrogel microbeads in the high concentration range of BSA (2000 µg/mL and above). Additionally, BSA molecules adsorbed onto the surfaces of cellulose/silk/Fe_3_O_4_ hydrogel microbeads at very high concentrations may interact with each other, leading to further adsorption. Such a phenomenon makes it challenging to apply the Langmuir model, which assumes monolayer adsorption, to describe the adsorption behavior [47].

The Langmuir model is an adsorption model for monolayer surface coverage with equal sorption activation energies, and there is no interaction between the adsorbates on the surface [48]. Furthermore, the dimensionless separation factor (R_L_) can be derived from the Langmuir parameters as follows:

$$R_{L}=\frac{1}{1+bC_{0}},$$

where b (mL/µg) is the Langmuir constant, and C_0_ (µg/mL) is the highest initial BSA concentration. The R_L_ values classify the isotherm type as irreversible (R_L_ = 0), favorable (0 < R_L_ < 1), linear (R_L_ = 1), or unfavorable (R_L_ > 1) [42]. The R_L_ values for the BSA adsorption on the cellulose/Fe_3_O_4_ and cellulose/silk/Fe_3_O_4_ hydrogel microbeads ranged from 0.1 to 0.5. This indicates that the adsorption environments were favorable.

The significantly enhanced initial adsorption rate ($k_{2}q_{e}^{2}$) and adsorption capacity (q_m_) for BSA of the cellulose/silk/Fe_3_O_4_ microbeads could be explained by the more negatively charged and hydrophobic surfaces resulting from the presence of silk, compared to the cellulose/Fe_3_O_4_ microbeads. The silk fibroin contains hydrophobic amino acids that could create strong electrostatic interactions through the carboxyl groups of silk and the amino groups of BSA [49]. Therefore, electrostatic attraction and the hydrophobic effect could be the major adsorption mechanisms of BSA on cellulose/silk/Fe_3_O_4_ hydrogel microbeads.

### 2.6. Release Profiles of BSA Adsorbed on Cellulose/Silk/Fe_3_O_4_ Microbeads

**Figure 9:**
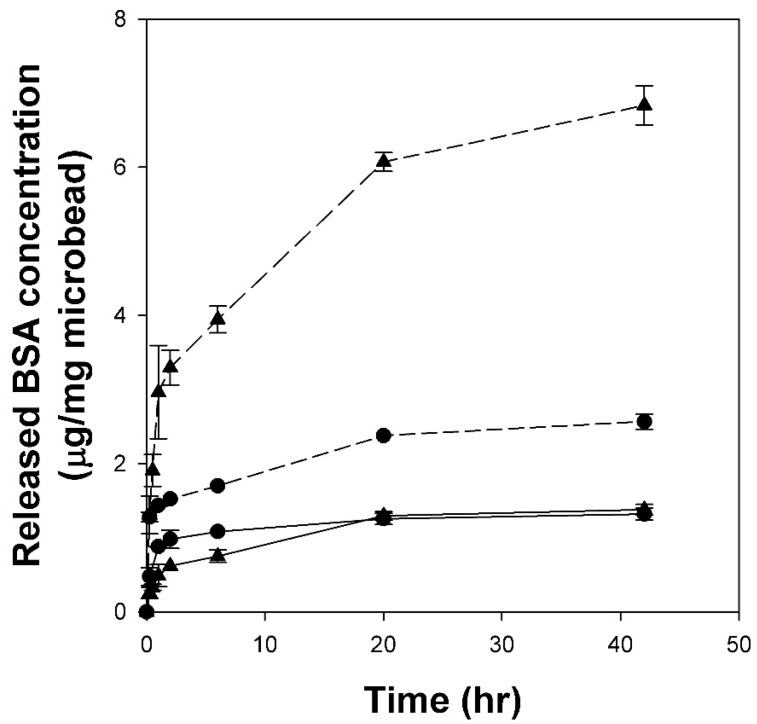
Cumulative release profiles of BSA from cellulose/silk/Fe_3_O_4_ microbeads at pH 2.2 and pH 7.4. Circle symbols (TBAH) and triangle symbols (TBPH) represent the solvents used to prepare the microbeads. Solid lines and dotted lines represent pH 2.2 and pH 7.4, respectively.

Stimulus-responsive microbeads have significant potential for biomedical applications owing to their ability to steadily deliver drugs to target sites for a prolonged duration. Specifically, pH-based release control systems play a crucial role in the development of oral delivery systems for protein drugs. We investigated whether cellulose/silk/Fe_3_O_4_ hydrogel microbeads can be used as a support that can release proteins through the pH difference between the gastrointestinal tract and the small intestine for oral protein drug delivery. To investigate the desorption characteristics of BSA adsorbed on cellulose/silk/Fe_3_O_4_ microbeads in response to pH changes, BSA-adsorbed microbeads were prepared, and desorption was performed at pH 2.2, simulating the stomach environment, and at pH 7.4, simulating the small-intestine environment (Figure 9). The testing environment was maintained at 37 °C, the average body temperature of humans. The release rate of BSA from the cellulose/silk/Fe_3_O_4_ microbeads was calculated by the first-order kinetics model (Table S4). Cellulose/silk/Fe_3_O_4_ microbeads prepared with TBAH exhibited an initial release rate of 2.2 µg/mg·h at pH 2.2, while at pH 7.4, the release rate was 5.4 µg/mg·h, indicating a 2.2-fold faster initial release at pH 7.4. In contrast, the cellulose/silk/Fe_3_O_4_ microbeads prepared with TBPH had an initial release rate of 1.0 µg/mg·h at pH 2.2, and at pH 7.4, the rate increased to 6.2 µg/mg·h, showing a 6.1-fold faster initial release at pH 7.4. These results demonstrate a positive outcome, indicating that cellulose/silk/Fe_3_O_4_ microbeads can control the initial release rate of BSA in response to pH changes. Examining the desorption trend over an extended period, the cellulose/silk/Fe_3_O_4_ microbeads maintained a consistently low level of BSA release at pH 2.2, with an overall release of approximately 1.3 µg/mg microbead even after 42 h. In contrast, at pH 7.4, the amount of released BSA gradually increased. For the cellulose/silk/Fe_3_O_4_ microbeads manufactured with TBAH and TBPH, the BSA release amounts after 42 h were 2.6 µg/mg microbead and 6.8 µg/mg microbead, respectively. These results signify that cellulose/silk/Fe_3_O_4_ microbeads can maintain a low desorption rate of BSA during passage through the stomach, thereby minimizing the loss of protein drugs. Simultaneously, they continuously desorb BSA in the small intestine, enabling the controlled release of protein drugs. These release behaviors were mainly due to the dependence of the release rate on the swelling pattern. It is conceivable that silk is protonated at pH 2.2, inhibiting the swelling of the microbeads and limiting the diffusion of BSA [50]. In particular, the cellulose/silk/Fe_3_O_4_ microbeads prepared with TBPH had a significant advantage over the cellulose/silk/Fe_3_O_4_ microbeads prepared with TBAH, showing a much greater difference in the rate and amount of BSA released owing to pH variation. This is likely because the cellulose/silk/Fe_3_O_4_ microbeads prepared with TBPH had somewhat rougher surfaces than those prepared with TBAH (Figure 2) and exhibited higher swelling ratios at neutral pH (Figure 3).

### 2.7. Cytotoxicity of Cellulose and Cellulose/Silk Composites

**Figure 10:**
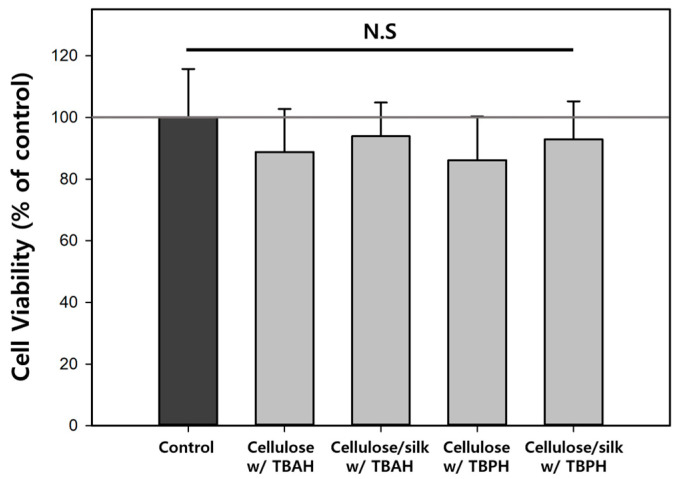
Effects of regenerated cellulose and cellulose/silk film on HaCaT cell viability. Cell viability was determined using an NR assay. One-way analysis of variance with Tukey’s test (*p* < 0.05); N.S: not significant.

pH-based release control systems have broad applications not only in oral delivery but also in treating infected burn wounds. HaCaT cells, being human epithelial keratinocytes, are well suited for assessing the biocompatibility of cellulose-based biopolymer materials when applied to the body. Therefore, the cytotoxicity of the cellulose and cellulose/silk composite films prepared using TBAH and TBPH was examined in HaCaT cells (Figure 10). There was no significant difference in the cell viability depending on the solvent used to dissolve the biopolymers. The cell viability values on the cellulose films regenerated with TBAH and TBPH were 88.8 and 86.1%, respectively. Similarly, for the regenerated cellulose/silk film, the cell viability values were 93.9 and 92.9% for each solvent. The cell viability values of all the biopolymer films were not significantly different from that of the control (one-way ANOVA, *p* < 0.05). This implied that TBAH or TBPH, which could potentially strongly bind and remain in the regenerated cellulose and cellulose/silk films, did not exhibit cytotoxicity. Cellulose and silk have been used in various applications, including tissue engineering and drug delivery, and their biocompatibilities are widely recognized [1,51]. Several previous studies have reported the low cytotoxicity of regenerated cellulose and silk fibroin films [52,53,54]. The cytotoxicity of Fe_3_O_4_ should also be evaluated for the application of cellulose/silk/Fe_3_O_4_ hydrogel microbeads in biomedicine. However, this was not considered in this study because of uniformity issues in the production of Fe_3_O_4_-containing films. According to Paramo et al., there was no impact on the cell viability even as the concentration of Fe_3_O_4_ in HepG2 cells increased up to 50 µg/mL, as measured by an MTS assay. Furthermore, there was no observed increase in the cell death rate by Fe_3_O_4_ related to LDH release from damaged cell membranes [55]. Wei et al. analyzed the cytotoxicity of Fe_3_O_4_ on a mouse fibroblast cell line (L929) through an MTT assay and discovered that, even at a concentration of 0.5 mg/mL, there was no reduction in the cell viability [56].

## 3. Conclusions

In this study, we developed a method to produce cellulose/Fe_3_O_4_ hydrogel microbeads via the sol–gel transition of a solvent-in-oil emulsion using various cellulose-dissolving solvents and soybean oil without the need for surfactants. TBAH and TBPH effectively dissolved cellulose at room temperature and facilitated Fe_3_O_4_ dispersion, enabling the easy separation of the cellulose/Fe_3_O_4_ hydrogel microbeads with an average diameter of approximately 15 µm. Notably, TBAH and TBPH co-dissolved cellulose and silk, allowing the manufacture of blended cellulose/silk/Fe_3_O_4_ hydrogel microbeads. This enabled the easy modification of the surface characteristics of the cellulose/Fe_3_O_4_ hydrogel microbeads, taking advantage of the excellent biocompatibility and degradability of silk, making it a suitable material for biomedical applications. Silk, owing to its strong interaction with proteins, possesses a remarkable protein adsorption capacity, making it a suitable material for protein drug delivery. Cellulose/silk/Fe_3_O_4_ hydrogel microbeads, with tunable surface charges and protein adsorption capabilities depending on the silk content, can serve as a support for protein delivery. Compared to cellulose/Fe_3_O_4_ hydrogel microbeads, cellulose/silk/Fe_3_O_4_ hydrogel microbeads, with a more negative surface charge, exhibited a 10 times higher adsorption capacity for the cationic dye CV. Additionally, because of the high protein adsorption capacity of silk, the cellulose/silk/Fe_3_O_4_ hydrogel microbeads exhibited an 18.1 times higher adsorption rate and an 8.5 times higher adsorption capacity for BSA than the cellulose/Fe_3_O_4_ hydrogel microbeads. The maximum q_e_ value for the BSA of the cellulose/silk/Fe_3_O_4_ hydrogel microbeads was 1643 mg/g, and this is significantly higher compared to those of previously reported cellulose-based microbeads. The adsorption characteristics of the BSA on the cellulose/silk/Fe_3_O_4_ hydrogel microbeads were interpreted using the pseudo-second-order kinetic and Langmuir isotherm models. Particularly, the cellulose/silk/Fe_3_O_4_ hydrogel microbeads produced with TBPH exhibited a much higher adsorption capacity for BSA than those produced with TBAH. The pH-dependent BSA release from the cellulose/silk/Fe_3_O_4_ hydrogel microbeads prepared with TBPH revealed a 6.1 times slower initial desorption rate and a 5.2 times lower desorption amount at pH 2.2 compared to at pH 7.4. Therefore, cellulose/silk/Fe_3_O_4_ hydrogel microbeads are considered suitable pH-responsive supports for orally administered protein pharmaceuticals. Cytotoxicity tests on cellulose and cellulose/silk composites regenerated with TBAH and TBPH yielded nontoxic results, suggesting the significant potential for the biomedical use of cellulose/silk/Fe_3_O_4_ hydrogel microbeads.

## 4. Materials and Methods

### 4.1. Materials

MCC, [Emim][Ac], iron oxide nanoparticles (Fe_3_O_4_, < 50 nm), sodium phosphate dibasic, BSA, penicillin–streptomycin solution (Hybri-Max^TM^), pepsin from porcine gastric mucosa (≥250 U/mg solid), and neutral red solution (0.33%) were purchased from Sigma-Aldrich (St. Louis, MO, USA). Lithium bromide (LiBr), zinc chloride (ZnCl_2_), sodium hydroxide, TU, potassium chloride, ethanol, isopropanol, *n*-hexane, and CV were purchased from Samchun Pure Chem. Co., Ltd. (Seoul, South Korea). TBAH (40% in water) and TBPH (40% in water) were purchased from Daejung Chemicals & Metals Co., Ltd. (Gyeonggi-do, South Korea) and Tokyo Chemical Industry Co., Ltd. (Chuo-ku, Tokyo, Japan), respectively. The Micro BCA^TM^ protein assay kit, Gibco^TM^ fetal bovine serum, and trypsin–EDTA solution were obtained from Thermo Fisher Scientific Inc. (Rockford, IL, USA). Soybean oil was purchased from Sajo Haepyo Co. Ltd. (Seoul, South Korea). All other chemicals used in this study were of analytical grade and used without further purification.

### 4.2. Silk-Degumming Process

Silk was obtained from *Bombyx mori* silkworm cocoons (Gyeongsangbuk-do, South Korea). To remove sericin and prevent contamination, the silkworm cocoons were treated by soaking in 0.5% (*w*/*w*) Na_2_CO_3_ at 90 °C for 1 h [25]. The silk fibroin was washed with deionized water and lyophilized.

### 4.3. Preparation of Cellulose/Fe_3_O_4_ Hydrogel Microbeads

Six different cellulose-dissolving solvents, including [Emim][Ac] and aqueous solutions of TBAH (40%), TBPH (40%), LiBr (60%), ZnCl_2_ (68%), and NaOH/TU (9.3%/7.4%), were used to develop cellulose/Fe_3_O_4_ hydrogel microbeads. MCC (4% *w*/*v*) was dissolved in the cellulose-dissolving solvents under the dissolution conditions outlined in Table 1, and then 0.5% (*w*/*v*) Fe_3_O_4_ was dispersed in cellulose solutions using a mortar for 20 min. After dissolution, the cellulose/Fe_3_O_4_/solvent mixture was added to soybean oil at a 10:1 ratio (oil:solvent mixture) and stirred at 800 rpm for 1.5 h. Afterward, excess ethanol was added to the emulsion and stirred for 1 h to regenerate the cellulose. When the cellulose was completely regenerated into the hydrogel form, the cellulose/Fe_3_O_4_ hydrogel microbeads were separated from the liquid phase using a neodymium magnet. The collected cellulose/Fe_3_O_4_ hydrogel microbeads were washed sequentially with *n*-hexane, isopropanol, ethanol, and distilled water. Finally, the prepared cellulose/Fe_3_O_4_ hydrogel microbeads were stored in HPLC-grade water.

### 4.4. Preparation of Cellulose/Silk/Fe_3_O_4_ Hydrogel Microbeads

To prepare cellulose/silk/Fe_3_O_4_ hydrogel microbeads, the degummed silk (0.25, 0.5, 1, 1.5, 2% *w*/*v*) was first dissolved in TBAH or TBPH at 45 °C for 20 min. After cooling the mixture to room temperature, 4% (*w*/*v*) cellulose was dissolved for 1.5 h at room temperature. Fe_3_O_4_ (0.5% *w*/*v*) was then dispersed in a cellulose/silk/solvent mixture using a mortar. After the dissolution process, the cellulose/silk/Fe_3_O_4_ hydrogel microbeads were prepared through the same procedure used to prepare the cellulose/Fe_3_O_4_ hydrogel microbeads mentioned above.

### 4.5. Characterization of Cellulose/Fe_3_O_4_-Based Hydrogel Microbeads

The morphology of the hydrogel microbeads was determined using optical microscopy after staining with a Congo red dye solution. The sizes of the cellulose-based hydrogel microbeads were measured using a particle size analyzer (Mastersizer 2000, Malvern, UK). The hydrogel microbeads were rapidly cooled at −80 °C and then freeze-dried under a vacuum for surface characterization. The freeze-dried microbeads were sputter-coated with platinum for observation using field-emission scanning electron microscopy (FE-SEM) (JSM6308; JEOL Ltd., Tokyo, Japan).

The swelling ratio of the cellulose-based/Fe_3_O_4_-based microbeads was investigated by comparing the weights in the wet and dry states. The microbeads were soaked in distilled water for 48 h and then filtered to determine the accurate weight of the hydrogel. The dry weight of the microbeads was measured after drying in a 60 °C oven for 24 h, until a constant weight was achieved. The swelling ratio was calculated with the following equation:

$$\mathrm{Swelling}\;\mathrm{ratio}=\frac{\left(W_{s}-W_{d}\right)}{W_{d}}\;\times \;100,$$

where $W_{s}$ and $W_{d}$ are the weights of swollen and dry microbeads, respectively.

The protein content of the silk-blended cellulose hydrogel microbeads was determined using a Micro BCA protein assay kit. The freeze-dried microbeads (4 mg) were precisely measured and dispersed in a solution comprising 0.8 mL of the BCA reagent and distilled water (1:1 ratio). After being heated for 1 h in a dry oven at 60 °C, the sample was slowly cooled to room temperature. The absorbance was measured at 562 nm using a spectrophotometer. To obtain the standard curve, degummed silk was dissolved in TBPH at 45 °C for 20 min. The prepared silk solution was placed into a dialysis bag (3.5 kDa Spectra/Por^®^ 6 dialysis membrane, Spectrum Labs. Inc., Rancho Dominguez, CA, USA), and dialysis was performed for four days in distilled water. The regenerated silk was collected, freeze-dried for 24 h, and used for the standard curve calculations.

The amount of silk released from the cellulose/silk/Fe_3_O_4_ microbeads after long-term storage (at 25 °C) and incubation at high temperatures (35 °C, 45 °C, and 55 °C) was measured to confirm their stability. Lyophilized microbeads were mixed in a 0.5 M citrate–phosphate buffer (pH 7.0) and shaken at 100 rpm. Samples were taken at five-day intervals to measure the presence of proteins using a Micro BCA assay.

Chemical stability of cellulose/silk/Fe_3_O_4_ microbeads was achieved by evaluating the degradation by pepsin. The pepsin was dispersed in citrate–phosphate buffer (pH 2.2) at a concentration of 3 mg/mL, simulating gastric fluid conditions. The cellulose/silk/Fe_3_O_4_ hydrogel microbeads were then mixed into the pepsin mixture and agitated for 2 h at 37 °C. Samples were taken to determine the digested protein content.

### 4.6. Dye Adsorption on Cellulose/Silk/Fe_3_O_4_ Hydrogel Microbeads

The CV solution was prepared by dissolving it in distilled water, with a concentration of 700 µg/mL, and adjusting the pH to 7.0. The 2.5 mg (based on dry weight) cellulose/silk/Fe_3_O_4_ hydrogel microbeads prepared with various silk contents (0.25, 0.5, 1, 1.5, and 2% *w*/*v*) were dispersed in 0.5 mL distilled water and then added to a 29.5 mL CV solution. The mixture was shaken continuously at 100 rpm at 25 °C for 24 h to facilitate dye adsorption. After the adsorption process reached equilibrium, the hydrogel microbeads were separated using a neodymium magnet. The remaining dye solution was centrifuged at 12,000 rpm for 2 min, and the CV concentration of the supernatant was measured using a spectrophotometer at 590 nm. The equilibrium adsorption capacity (q_e_) of the cellulose/silk/Fe_3_O_4_ hydrogel microbeads was calculated as follows:

$$q_{e}=\frac{\left(C_{0}-C_{e}\right)}{W}\;\times \;V,$$

where C_0_ is the initial concentration of the CV dye solution (µg/mL); C_e_ is the equilibrium concentration of the CV dye solution (µg/mL); V is the total volume of the adsorption solution (mL); and W is the dry weight of the microbeads (mg).

### 4.7. Protein Adsorption and Release Study on Cellulose/Silk/Fe_3_O_4_ Hydrogel Microbeads

For the adsorption of BSA on cellulose/silk/Fe_3_O_4_ hydrogel microbeads, 2.5 mg (based on dry weight) microbeads were dispersed in 0.5 mL of 0.1 M phosphate buffer (pH 7.0) and then mixed with 24.5 mL of BSA solution at various concentrations. The BSA concentrations were determined using a Micro BCA protein assay kit. In the kinetics study, cellulose/Fe_3_O_4_ hydrogel microbeads and cellulose/silk/Fe_3_O_4_ hydrogel microbeads were shaken for up to 6 h in BSA solutions at concentrations of 100 and 500 µg/mL, respectively, to investigate the impact of the contact time. The adsorption was performed in a shaking incubator at 25 °C and 100 rpm. The amount of loaded BSA was determined indirectly by collecting 40 µL of the BSA solution at several time intervals and measuring the decreases from the initial concentration. In the isotherm study, BSA solutions were prepared at various concentrations ranging from 50 to 650 µg/mL for cellulose/Fe_3_O_4_ hydrogel microbeads and from 100 to 2500 µg/mL for cellulose/silk/Fe_3_O_4_ hydrogel microbeads. The adsorption conditions were identical to those used in the kinetics study. At equilibrium, the hydrogel microbeads were separated using a neodymium magnet and then centrifuged. The adsorbed BSA content was calculated indirectly by measuring the remaining BSA content in the supernatant.

In vitro BSA release studies were conducted individually in different pH release media (pH 2.2 and 7.4) using a wide range of citrate–phosphate buffers (0.5 M). The cellulose/silk/Fe_3_O_4_ microbeads used for desorption were prepared through the adsorption of 500 µg/mL of the BSA solution until equilibrium. The weakly bound BSA and adsorption medium were removed by washing with the phosphate buffer thrice. Finally, the BSA-loaded cellulose/silk/Fe_3_O_4_ hydrogel microbeads were lyophilized. The prepared BSA-loaded microbeads (8 mg) were measured and immersed in 4 mL of the release medium. The release study was performed in a shaking incubator at 37 °C and 100 rpm. The amount of released BSA was measured periodically after centrifugation.

### 4.8. Cytotoxicity of Regenerated Cellulose and Cellulose/Silk Film

Cellulose and cellulose/silk were dissolved in TBAH or TBPH and regenerated into film forms. After washing several times with distilled water, the films were completely dried in a 40 °C dry oven. Each film was trimmed to fit into a 96-well plate, sterilized with EtOH, and dried again. Gelatin was diluted to 0.5% using an autoclaved PBS buffer and added to each well, including the control. After incubating for 30 min in a 37 °C incubator, the gelatin solution was removed, and the prepared films were attached. HaCaT cells were cultured in Dulbecco’s modified Eagle’s medium with 10% fetal bovine serum, penicillin (100,000 U/L), and streptomycin (100 mg/L). The cells were seeded into 96-well plates at a density of 6500 cells per well and cultivated in an incubator at 37 °C in a humidified atmosphere of 5% CO_2_. Once the cells had grown, the medium was removed, and the live cells were stained with a neutral red solution for 3 h. Subsequently, 100 µL of an NR desorb solution was applied, and the plate was shaken for 20 min. The supernatants were then transferred to new wells, and their absorbances were measured at 540 nm using a Mobi microplate reader (MicroDigital Co., Ltd., Gyeonggi-do, South Korea).

The cell viability (%) was calculated using the following equation:

$$\mathrm{Cell}\;\mathrm{viability}\;(\%)=\frac{A_{s}\;-A_{b}}{A_{c}\;-A_{b}}\;\times \;100,$$

where A_s_ is the absorbance of the cellulose-based film, and A_b_ and A_c_ represent the absorbance of the blank without cells and that of the blank with cells, respectively.

## Figures and Tables

**Table 1:** Dissolution conditions of cellulose and average diameters of cellulose/Fe_3_O_4_ hydrogel microbeads prepared using various cellulose-dissolving solvents.

Dissolution Conditions			Average Diameter of Cellulose/Fe_3_O_4_ Microbeads (µm)
Solvent	Temperature (°C)	Time (min)	
[Emim][Ac]	100	120	48.8
LiBr (60%)	100	60	140.3
ZnCl_2_ (68%)	80	60	58.3
TBAH (40%)	RT *	90	14.6
TBPH (40%)	RT	90	17.5
NaOH/thiourea (9.3%/7.4%)	4	20	80.7

* Room temperature.

**Table 2:** Kinetic parameters of BSA adsorption on cellulose-based hydrogel microbeads.

Solvent	Silk Content (%)	Pseudo-Second-Order Model			q_e, exp._ (mg/g)
		k_2_ (×10^−3^ g/mg·h)	q_e, cal._ (mg/g)	R^2^	
TBAH	0	9.46	201.8	0.999	184.0
	1	25.33	524.8	0.999	507.6
TBPH	0	8.11	225.9	0.996	207.9
	1	9.82	611.4	0.997	569.7

**Table 3:** Isotherm parameters of BSA adsorption on cellulose/silk/Fe_3_O_4_ hydrogel microbeads.

Solvent	Silk Content (%)	Langmuir Model			q_e, exp._ (mg/g)
		b (×10^−3^ L/mg)	q_m_ (mg/g)	R^2^	
TBAH	0	12.65	220.4	0.973	185.4
	1	1.34	1378.0	0.993	1095.2
TBPH	0	14.88	228.5	0.985	194.3
	1	0.59	3142.0	0.996	1643.1

## Data Availability

The original contributions presented in the study are included in the article/Supplementary Material, further inquiries can be directed to the corresponding authors.

## Supplementary Materials

The following supporting information can be downloaded at https://www.mdpi.com/article/10.3390/gels10030200/s1, Table S1: Kinetic parameters of BSA adsorption on cellulose-based hydrogel microbeads; Table S2: Comparison of adsorption capacities of cellulose-based microbeads for BSA; Table S3: Isotherm parameters of BSA adsorption on cellulose/silk/Fe_3_O_4_ hydrogel microbeads; Table S4: Kinetic parameters of BSA release from cellulose/silk/Fe_3_O_4_ microbeads; Figure S1: FT-IR spectra of MCC (a), regenerated cellulose with TBAH (b), regenerated cellulose/silk with TBAH (c), regenerated cellulose with TBPH (d), regenerated cellulose/silk with TBPH (e), and degummed silk (f); Figure S2: Storage stability of cellulose/silk/Fe_3_O_4_ hydrogel microbeads at 25 °C. Circles (TBAH) and triangles (TBPH) represent the solvents used to prepare the microbeads. The contents of cellulose, silk, and Fe_3_O_4_ in the microbead-preparing solution were 4, 1, and 0.5%, respectively; Figure S3: Thermal stability of cellulose/silk/Fe_3_O_4_ hydrogel microbeads after 24 h incubation. Black, gray, and dark-gray bars represent incubation temperatures of 35 °C, 45 °C, and 55 °C, respectively. The contents of cellulose, silk, and Fe_3_O_4_ in the microbead-preparing solution were 4%, 1%, and 0.5%, respectively.
