# Supplementary material for: pH-Responsive Cellulose/Silk/Fe3O4 Hydrogel Microbeads Designed for Biomedical Applications
Source: Gels. 2024 Mar 16;10(3):200. doi: 10.3390/gels10030200 (PMC10969998; doi:10.3390/gels10030200)
Supplement: Supplementary file 1 [file gels-10-00200-s001.zip › gels-2896756-supplementary.pdf]

pH-Responsive cellulose/silk/Fe_3O_4 hydrogel microbeads designed for biomedical applications

Seung Hyeon Weon†, Yuhyeon Na†, Jiwoo Han, Jeong Woo Lee, Hyung Joo Kim, Saerom Park* and Sang Hyun Lee*

Department of Biological Engineering, Konkuk University, Seoul 05029, Republic of Korea.

***Corresponding authors:**

Saerom Park

Tel.: +82-10-4038-4656

E-mail: angel4y@naver.com

Sang Hyun Lee

Tel.: +82-2-2049-6269

Fax: +82-2-457-8895

E-mail: sanghlee@konkuk.ac.kr

† Seung Hyeon Weon and Yuhyeon Na equally contributed to this study.

Table S1. Kinetic parameters of BSA adsorption on cellulose-based hydrogel microbeads.

Solvent	Silk content (%)	Pseudo-first-order model			$q_{e, exp.}$ (mg/g)
		k_1 (1/min)	$q_{e, cal.}$ (mg/g)	R^2	
TBAH	0	1.57	250.3	0.945	184.0
	1	-1.93	14.0	0.691	507.6
TBPH	0	-0.48	23.9	0.603	207.9
	1	-2.24	12.0	0.799	569.7

Table S2. Comparison of adsorption capacities of cellulose-based microbeads for BSA.

Adsorbent	Adsorbate	q_{max} (mg/g)	Reference
Cellulose beads	BSA	204.6	[43]
Cellulose/agarose composite microspheres	BSA	307.6	[44]
Diethylaminoethyl-modified cellulose microspheres	BSA	409.5	[45]
Cellulose/silk/Fe_3O_4 hydrogel microbeads	BSA	1643.1	This work

Table S3. Isotherm parameters of BSA adsorption on cellulose/silk/Fe_3O_4 hydrogel microbeads.

Solvent	Silk Content (%)	Freundlich Model			Elovich Model		
		k_f (mg/g)	n	R^2	k_E (× 10^{-2} L/mg)	q_m (mg/g)	R^2
TBAH	0	40.46	4.02	0.848	17.80	58.3	0.780
	1	14.20	1.75	0.985	0.25	810.5	0.941
TBPH	0	37.45	3.64	0.872	14.75	66.1	0.792
	1	7.38	1.38	0.973	0.09	2244.8	0.755

Table S4. Kinetic parameters of BSA release from cellulose/silk/Fe_3O_4 microbeads.

Solvent	pH	Zero-Order Model		First-Order Model		Higuchi Model	
		K_0	R^2	K_1	R^2	K_H	R^2
TBAH	2.2	0.19	0.628	0.002	0.813	1.44	0.764
	7.4	0.29	0.879	0.039	0.897	2.63	0.977
TBPH	2.2	0.33	0.813	0.004	0.824	2.44	0.941
	7.4	0.10	0.781	0.035	0.957	9.39	0.925

Zero-order model: $Q_t = K_0 \cdot t$,

First-order model: $logC = logC_0 - \frac{K_1 t}{2.303}$,

Higuchi model: $Q_t = K_H \cdot t^{0.5}$,

where Q_t is the cumulative amount of released protein; K_0 is the zero-order rate constant; C is the percentage of remaining protein; K_1 is the first-order rate constant; K_H is the Higuchi constant; and t is time (h).

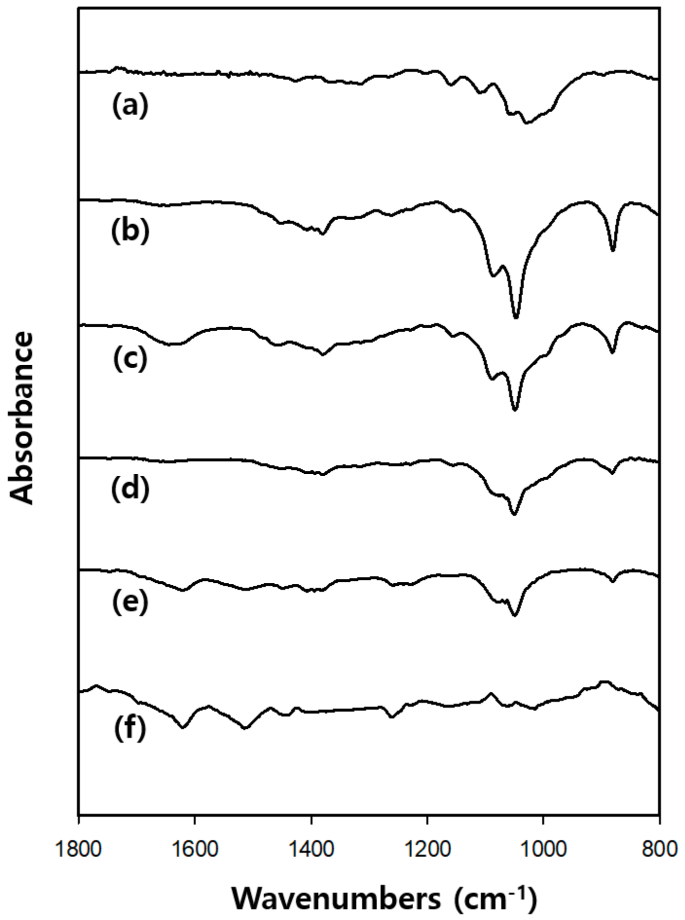

Figure S1. FT-IR spectra of MCC (a), regenerated cellulose with TBAH (b), regenerated cellulose/silk with TBAH (c), regenerated cellulose with TBPH (d), regenerated cellulose/silk with TBPH (e), and degummed silk (f).

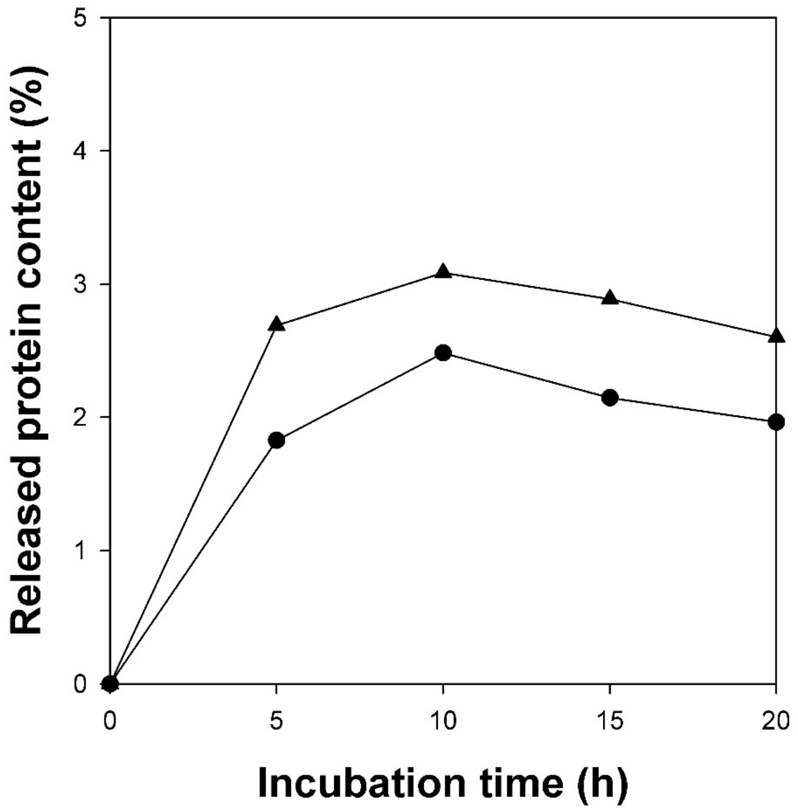

Figure S2. Storage stability of cellulose/silk/Fe_3O_4 hydrogel microbeads at 25°C. Circle symbols (TBAH) and triangle symbols (TBPH) represent the solvents used to prepare microbeads. The contents of cellulose, silk, and Fe_3O_4 in the microbead-preparing solution were 4%, 1%, and 0.5%, respectively.

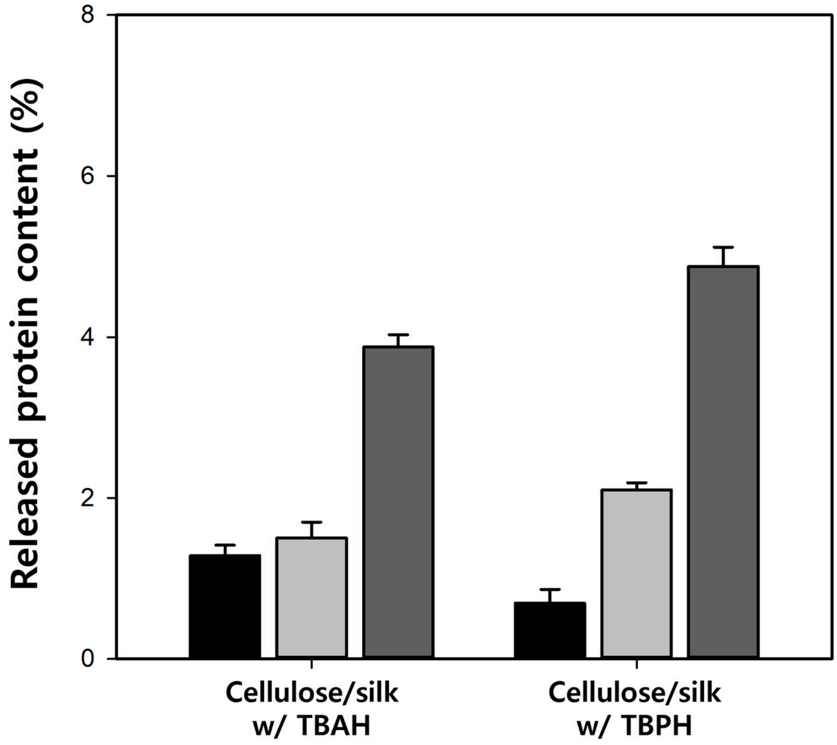

Figure S3. Thermal stability of cellulose/silk/Fe_3O_4 hydrogel microbeads after 24 h incubation. Black, gray, and dark gray bars represent the incubation temperatures of 35°C, 45°C, and 55°C, respectively. The contents of cellulose, silk, and Fe_3O_4 in the microbead-preparing solution were 4%, 1%, and 0.5%, respectively.
